# Assessment of the tocolytic nifedipine in preclinical primary models of preterm birth

**DOI:** 10.1038/s41598-023-31077-x

**Published:** 2023-04-06

**Authors:** Bridget M. Arman, Natalie K. Binder, Natasha de Alwis, Sally Beard, Danielle A. Debruin, Alan Hayes, Stephen Tong, Tu’uhevaha J. Kaitu’u-Lino, Natalie J. Hannan

**Affiliations:** 1 Therapeutics Discovery and Vascular Function Group, Department of Obstetrics and Gynaecology, University of Melbourne, Mercy Hospital for Women, 163 Studley Rd, Heidelberg, Victoria 3084 Australia; 2 Mercy Perinatal, Mercy Hospital for Women, Heidelberg, 3084 Australia; 3 Institute for Health and Sport, Victoria University, Melbourne, Victoria 3000 Australia; 4 Australian Institute for Musculoskeletal Science, Victoria University, St AlbansVictoria, 3021 Australia; 5 Department of Medicine—Western Health, Melbourne Medical School, University of Melbourne, St Albans, Victoria 3021 Australia

**Keywords:** Preclinical research, Medical research, Reproductive disorders

## Abstract

Spontaneous preterm birth is the leading cause of perinatal morbidity and mortality. Tocolytics are drugs used in cases of imminent preterm birth to inhibit uterine contractions. Nifedipine is a calcium channel blocking agent used to delay threatened spontaneous preterm birth, however, has limited efficacy and lacks preclinical data regarding mechanisms of action. It is unknown if nifedipine affects the pro-inflammatory environment associated with preterm labour pathophysiology and we hypothesise nifedipine only targets myometrial contraction rather than also mitigating inflammation. We assessed anti-inflammatory and anti-contractile effects of nifedipine on human myometrium using in vitro and ex vivo techniques, and a mouse model of preterm birth. We show that nifedipine treatment inhibited contractions in myometrial in vitro contraction assays (P = 0.004 vs. vehicle control) and potently blocked spontaneous and oxytocin-induced contractions in ex vivo myometrial tissue in muscle myography studies (P = 0.01 vs. baseline). Nifedipine treatment did not reduce gene expression or protein secretion of pro-inflammatory cytokines in either cultured myometrial cells or ex vivo tissues. Although nifedipine could delay preterm birth in some mice, this was not consistent in all dams and was overall not statistically significant. Our data suggests nifedipine does not modulate preterm birth via inflammatory pathways in the myometrium, and this may account for its limited clinical efficacy.

## Introduction

Globally, prematurity is the leading cause of neonatal morbidity and mortality, with an estimated 15 million babies born preterm each year^1^. Prolongation of pregnancies with threatened spontaneous preterm delivery is vital, particularly in very early gestation, as each completed week in utero corresponds to significantly improved fetal outcomes^2^. Ideally, the aim of inhibiting spontaneous preterm birth is to prolong pregnancies until they are closer to term. However this is rarely attainable with the current panel of therapeutics available.

Spontaneous preterm labour is primarily treated with drugs, known as tocolytics, that inhibit uterine contractions. There are several choices of tocolytics, with each targeting a different mechanism of uterine contraction, however there is no strong evidence that any tocolytic improves neonatal outcomes^3^. Realistically, the administration of these drugs only delays delivery with enough time for corticosteroid treatment for fetal lung maturation, magnesium sulphate for fetal neuroprotection (in some instances), antibiotics in the case of infection, and for transport of the patient to an appropriate tertiary hospital.

Nifedipine is one of the most widely used tocolytics. Conventionally used as an anti-hypertensive agent, it was repurposed as a tocolytic in the 1980s due to its calcium channel blocking abilities^4^. Nifedipine is an L-type voltage-gated calcium channel antagonist. It inhibits the influx of extracellular calcium into smooth muscle cells, preventing calcium-dependent contractions^5^. Despite nifedipine being considered the standard course of treatment for preterm labour, clinical trials evidence supporting the benefit of nifedipine is limited^6^.

Before transition to clinical use in the 1980s, there was only a small number of preclinical studies investigating nifedipine as a tocolytic that preceded trials in patients^4,7,8^. These few studies were limited, assessing nifedipine’s ability to reduce uterine contractile activity, therefore much remains unknown about the mechanism of action nifedipine works through. Moreover, there is a lack of human in vitro data to determine how nifedipine functions at the cellular level in the mitigation of preterm labour. Importantly, it is unknown if nifedipine has beneficial actions beyond calcium channel inhibition.

Nifedipine, as well as other tocolytics, work by supressing myometrial contractility, the consequence rather than the cause of preterm labour. It is unknown whether these tocolytics have an impact on the upstream inflammatory mediators implicated in the pathophysiology of preterm labour. Importantly, the initiation of labour is complex, involving a milieu of uterine pro-inflammatory cytokine and chemokine signalling at both term and preterm gestations^9^. Normal healthy labour is characterised by leukocyte influx, release of pro-inflammatory cytokines such as interleukin (IL)-1B, IL-6, CXC motif chemokine ligand (CXCL)-8 and tumor necrosis factor (TNF), and a decrease of anti-inflammatory agents in the uterus^9^. Spontaneous preterm labour is caused by similar inflammatory processes but at a pathological level of inflammation^10,11^. Pathogens or danger signals bind to and activate toll-like receptors in the uterus which triggers a downstream pro-inflammatory cascade culminating in activation of myometrial contractility, rupture of membranes, and cervical remodelling, ultimately leading to preterm labour^10–12^. It is unknown if nifedipine has anti-inflammatory effects that can act on mitigating these upstream events. Such knowledge could be used to increase the efficacy of tocolytic compounds. While it is believed that the primary mechanism of nifedipine in the uterus is to prevent calcium-dependent muscle contractions, there is evidence in non-gestational tissues that nifedipine may exert anti-inflammatory and cytoprotective actions beyond inhibition of calcium channels^13,14^. Therefore, we aimed to test whether nifedipine demonstrated similar actions in the myometrium.

The current study employed an innovative pipeline of human in vitro and ex vivo functional models of myometrial contraction and inflammation, and a mouse model of preterm birth, to assess the actions of nifedipine on myometrium.

## Results

### Human myometrial tissue strip contractility

**Figure 1:**
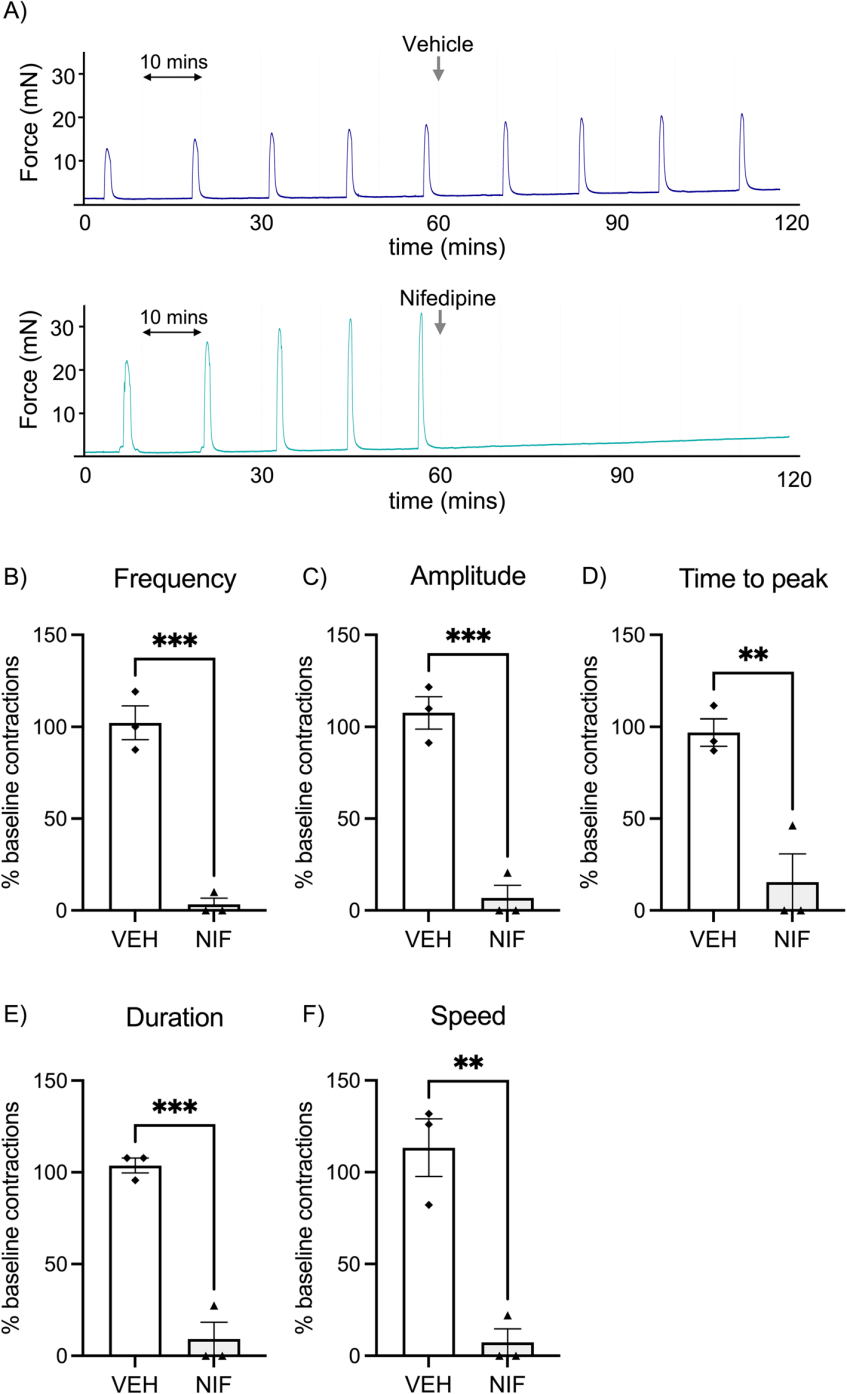
Myograph data showing anti-contractile effects of nifedipine (0.1 µM) on strips of human pregnant non-labouring myometrium ex vivo. (**A**) Representative trace in the upper panel showing that vehicle control (VEH) has no effect on established spontaneous contractions. In the lower panel, nifedipine (NIF) diminishes the established pre-treatment contractions. The y-axis is the measured force (millinewtons; mN). The entire initial 120 min equilibration period is not shown in these representative traces. (**B–F**) Nifedipine significantly reduces contraction frequency (**B**), amplitude (**C**), time to peak (**D**), contraction duration (**E**), and contraction speed (**F**) (*n* = 3 patients). Data are presented as percentage of baseline (pre-treatment) contractility. Each point represents the mean of duplicates. Data were assessed for statistical differences using t tests. The error bars represent SEM, ** indicates P < 0.01, and *** indicates P<0.001.

We first assessed ex vivo myometrial tissue contractility via organ bath myography to determine the effect of nifedipine on non-labouring human myometrial tissue (collected at caesarean section; *n* = 3). Nifedipine treatment significantly inhibited baseline spontaneous contraction frequency in myometrial strips (Fig. 1A lower panel, B; P = 0.001). Where contractions did occur in the post-treatment period after nifedipine administration, the peaks were significantly reduced in amplitude, time to peak, duration, and speed compared with vehicle (Fig. 1C–F, all P < 0.01). Vehicle control treatment had no effect on contraction frequency (Fig. 1A upper panel), nor on other measures of contractility (amplitude, time to peak, duration of single contractions, and speed; Supplementary Fig. S1). Importantly, we assessed myometrial contraction at the end of assessment using high potassium solution which causes maximal influx of calcium ions into the cells and is used to test tissue integrity. Tissue was responsive to challenge at the completion of the experiment, indicating the tissue was functionally active (not fatigued) after the 4–5 h in the myograph chambers (Supplementary Fig. S2).

**Figure 2:**
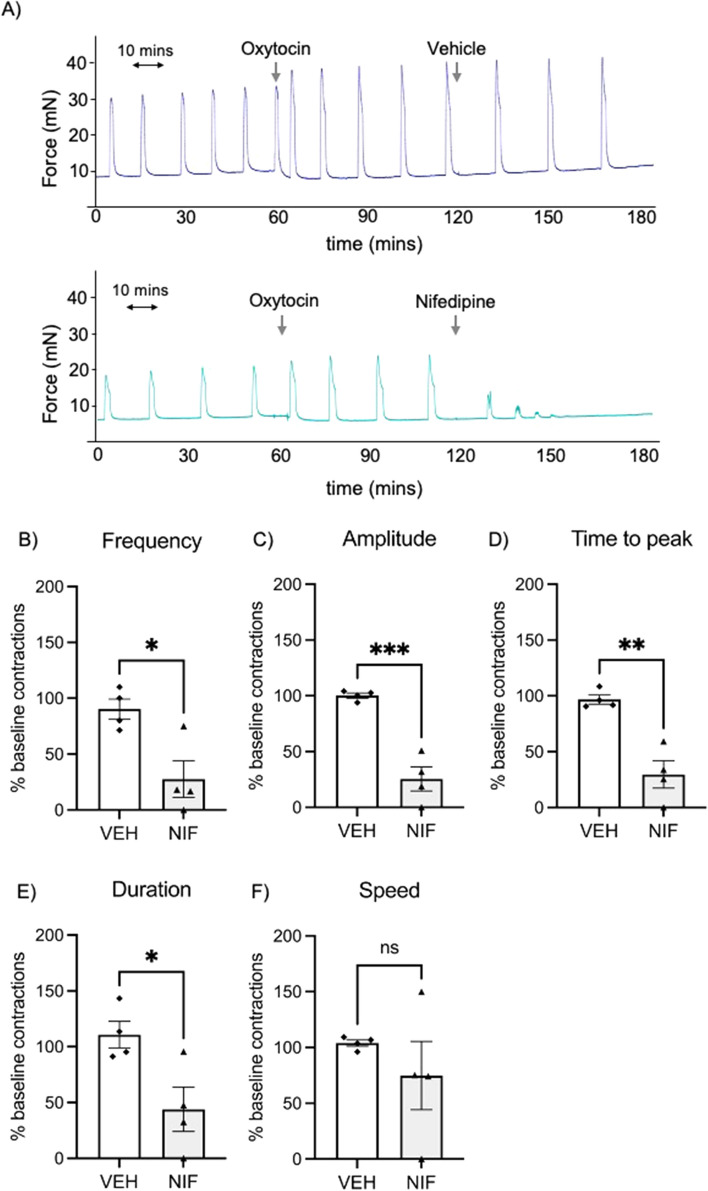
Myograph data showing anti-contractile effects of nifedipine (0.1 µM) on strips of human pregnant non-labouring myometrium ex vivo after incubation with oxytocin (1 nM). (**A**) Representative traces showing that vehicle control (VEH) does not affect frequency of established oxytocin-induced contractions, but nifedipine (NIF) significantly reduces the contraction frequency. The y-axis presents the measured force (millinewtons; mN). The entire initial 120 min equilibration period is not shown in these representative traces. (**B–F**) In the presence of oxytocin, nifedipine significantly reduces contraction frequency (**B**), amplitude (**C**), time to peak (**D**), and contraction duration (**E**) but not contraction speed (**F**) (*n* = 4 patients). Each point represents the mean of duplicates. Data were assessed for statistical differences using t tests. The error bars represent SEM, * indicates P < 0.05, ** indicates P<0.01, *** indicates P<0.001, and ns denotes no statistical difference.

Myometrial strips collected from another cohort of patients (*n* = 4) were treated with oxytocin in the myograph tissue baths to further induce contractions. Still in the presence of oxytocin, the myometrial strips were treated with either vehicle control or nifedipine for one hour. Vehicle control did not alter the frequency of oxytocin-induced contractions (Fig. 2A upper panel), but treatment with nifedipine reduced them (Fig. 2A lower panel, B; P = 0.01). Similarly, nifedipine significantly reduced the other measures of contractility (amplitude, time to peak and duration) compared with vehicle control (Fig. 2B–E, all P < 0.05). There was no statistically significant difference in speed of contractions between vehicle- and nifedipine-treated tissue as the variation within the nifedipine-treated samples was high (Fig. 2F).

### Human myometrial cell contraction assay

We then assessed whether nifedipine inhibits myometrial contraction in the collagen-myometrial cell contraction assay over a longer period of time. We first established that the basal level of contraction inherent to the cells was a 6.3 ± 1.0% decrease in collagen gel disc size after 48 h, compared to the baseline gel size at 0 h (Supplementary Fig. S3). Treatment with nifedipine alone did not alter the rate of these basal contractions (Supplementary Fig. S4). We next examined whether nifedipine treatment altered collagen-cell contractility within a TNF/LPS-stimulated inflammatory environment. Treatment with TNF/LPS increased collagen-cell disc contractility compared to the basal contraction rate of control-treated cells, with a mean 13.6 ± 0.9% decrease in gel size 48 h after treatment (Supplementary Fig. S3; P = 0.01). Co-treatment with nifedipine demonstrated inhibition of TNF/LPS induced contraction (Supplementary Fig. S3; P = 0.004), maintaining a level of contraction similar to the basal vehicle control conditions at 48 h (Supplementary Fig. S3; P = 0.66).

### Human myometrial cell inflammatory response

After establishing that nifedipine inhibited contraction, we next assessed whether nifedipine altered inflammatory pathways. Human myometrial cells (cell line) were stimulated with pro-inflammatory agents TNF or LPS.

**Figure 3:**
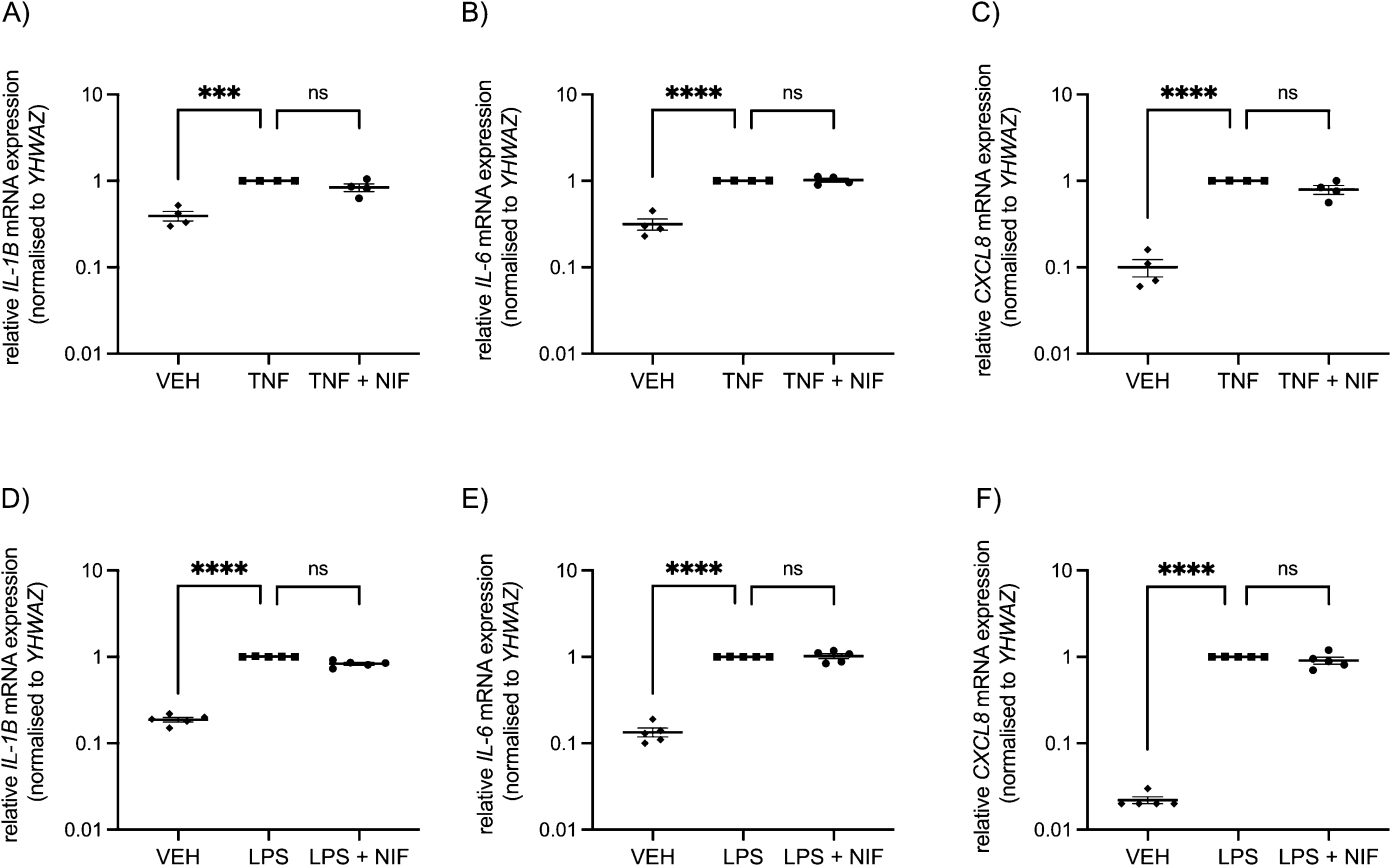
Gene expression of pro-inflammatory cytokines by human myometrial cells after treatment with vehicle control (VEH), TNF, LPS, and nifedipine (NIF). Treatment of myometrial cells with (**A–C**) TNF (0.1 ng/ml) and (**D–F**) LPS (100 ng/ml) induces an increase in pro-inflammatory mRNA expression of *IL-1B*, *IL-6*, and *CXCL8.* Addition of nifedipine (10 µM) does not reduce this upregulation. Data are presented as fold change calculated relative to that of TNF-treated cells (**A–C**) or LPS-treated cells (**D–F**). Treatments were performed in duplicate and individual data points represent the mean of those technical replicates (*n* = 4 experiments (**A–C**) and *n* = 5 experiments (**D–F**)). Data were assessed for statistical differences using one-way ANOVA followed by Dunnett’s multiple comparisons performed on the deltaCt (Ct of the gene of interest subtracted from the Ct of the *YHWAZ* reference gene). The error bars represent SEM, *** indicates P < 0.001, **** indicates P < 0.0001, and ns indicates no statistical difference.

Treatment with TNF caused a clear upregulation in expression of pro-inflammatory cytokine genes: *IL-1B* (P < 0.001), *IL-6* (P < 0.0001), and *CXCL8* (P < 0.0001) compared with vehicle control treatment (Fig. 3A–C). However, co-treatment with nifedipine did not attenuate this elevated gene expression (Fig. 3A–C).

Similarly, stimulation of myometrial cells with LPS induced a significant elevation of *IL-1B, IL-6*, and *CXCL8* mRNA transcripts compared with vehicle control (P < 0.0001, Fig. 3D–F). Co-treatment with nifedipine did not inhibit this increased expression (Fig. 3D–F). Cell viability was not affected by any of these treatments (Supplementary Fig. S5).

**Figure 4:**
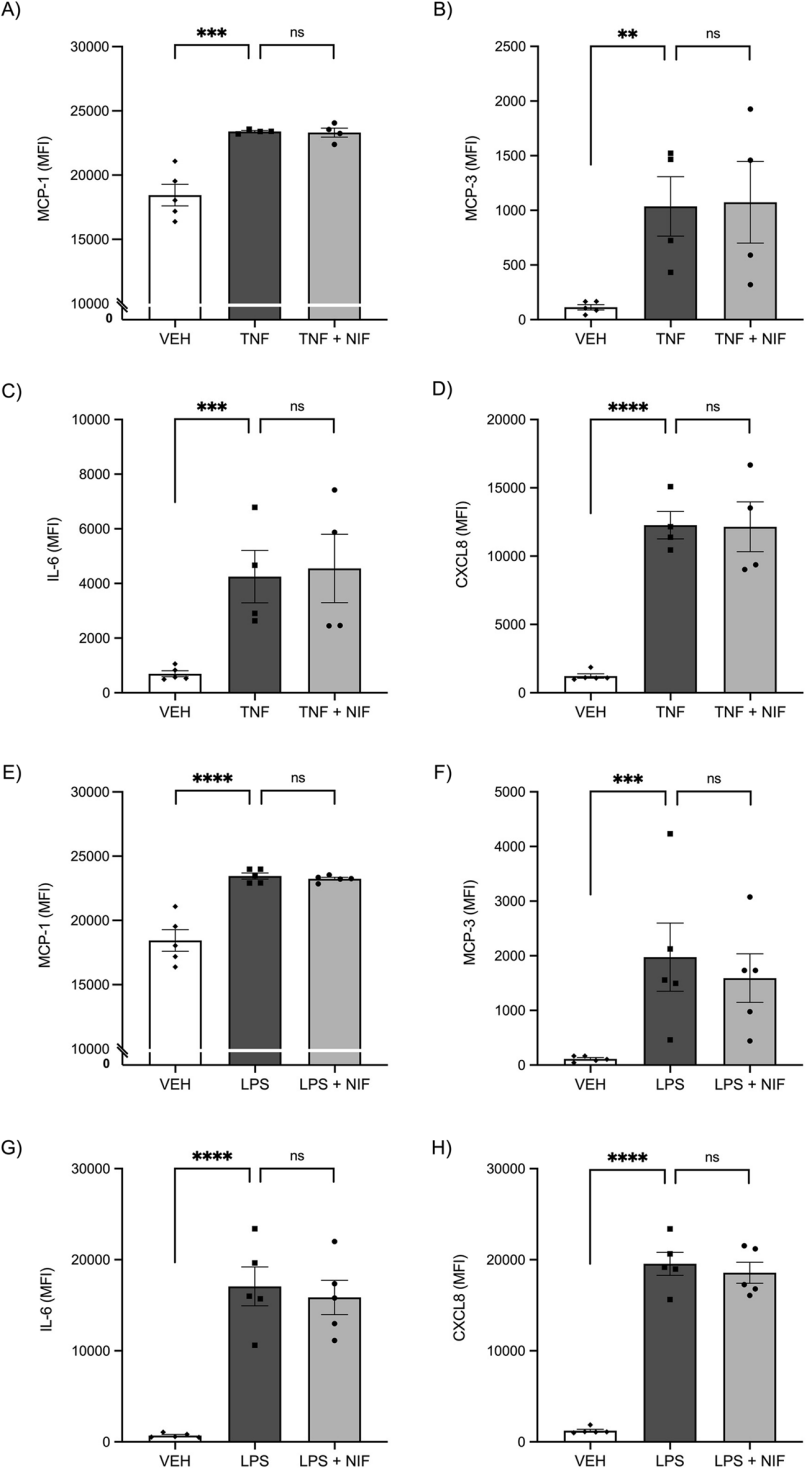
Pro-inflammatory cytokines and chemokines secreted by human myometrial cells were assessed in media collected after treatment with vehicle control (VEH), TNF, LPS, and nifedipine (NIF). Treatment of myometrial cells with (**A–D**) TNF (0.1 ng/ml) and (**E–H**) LPS (100 ng/ml) induces a significant increase in production and release into culture media of pro-inflammatory cytokines and chemokines MCP-1, MCP-3, IL-6, and CXCL8 and co-treatment with nifedipine (10 µM) does not reduce this increased expression. Data is expressed as the median fluorescence intensity of technical replicates as measured by Luminex assay. Statistical analysis was performed on the log2 transformation of the fluorescence intensity using an ANOVA followed by Tukey’s multiple comparisons. Treatments were performed in duplicate and individual data points represent the mean of those technical replicates (*n* = 4 experiments). The error bars represent SEM, ** indicates P < 0.01, *** indicates P < 0.001, **** indicates P < 0.0001, and ns indicates no statistical difference.

We next assessed the secretion of cytokines into the conditioned media from the treated myometrial cells. Treatment with either TNF (Fig. 4A–D) or LPS (Fig. 4E–H) induced a significant increase in secretion of soluble MCP-1, MCP-3, IL-6, and CXCL8 from the cells compared with control (all P < 0.05). However, there was no difference in secretion of pro-inflammatory cytokines with nifedipine treatment (Fig. 4). IL-1B concentrations were below the level of detection of the multiplex assay.

### Human myometrial tissue inflammatory response

**Figure 5:**
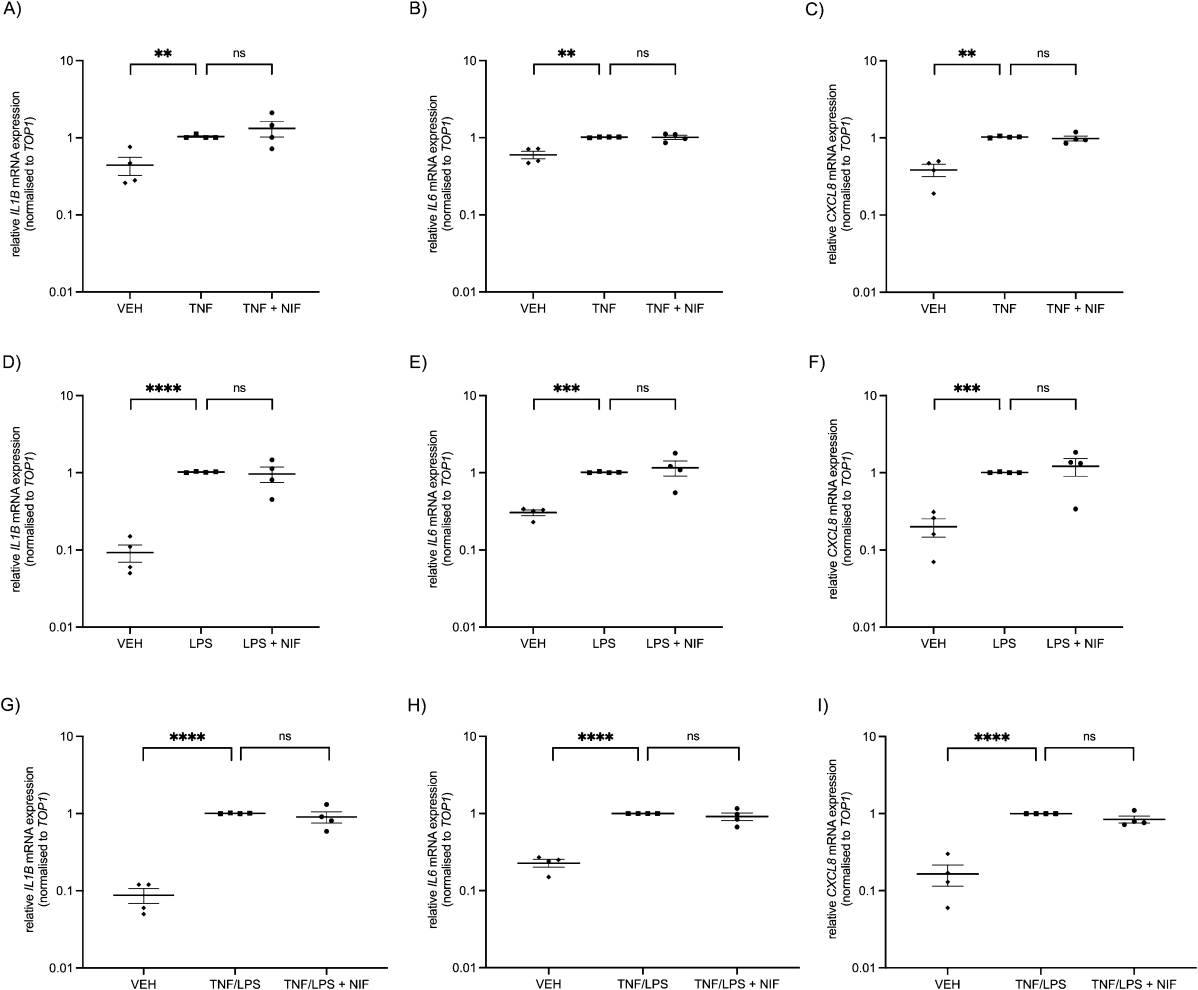
Gene expression of pro-inflammatory cytokines by human myometrial tissue after treatment with vehicle control (VEH), TNF, LPS and nifedipine (NIF). Treatment of myometrial tissue pieces with (**A–C**) TNF (1 ng/ml), (**D–F**) LPS (5 ng/ml) or (**G–I**) concurrent TNF and LPS treatment increases pro-inflammatory mRNA expression of *IL-1B*, *IL-6*, and *CXCL8.* Addition of nifedipine (10 µM) does not reduce this upregulation. Data are presented as fold change calculated relative to that of TNF-treated tissue (**A–C**), LPS-treated tissue (**D–F**), or combined TNF/LPS-treated tissue (**G–I**). Treatments were performed in duplicate and individual data points represent the mean of those technical replicates (*n* = 4 experiments). One-way ANOVA followed by Dunnett’s multiple comparisons was performed on the deltaCt (Ct of the gene of interest subtracted from the Ct of the *TOP1* reference gene). The error bars represent SEM, ** indicates P < 0.01, *** indicates P < 0.001, **** indicates P < 0.0001, and ns indicates no statistical difference.

We next investigated if nifedipine treatment inhibits the inflammatory response in cultured whole human myometrial tissue. Treatment with TNF increased *IL-1B* (P = 0.003), *IL-6* (P = 0.008) and *CXCL8* (P = 0.002) gene expression compared with vehicle control-treatment (Fig. 5A–C). Addition of nifedipine did not reduce this increased expression of *IL-1B*, *IL-6,* or *CXCL8* (Fig. 5A–C).

Treatment of myometrial tissue with LPS induced an increase of *IL-1B* (P < 0.0001)*, IL-6* (P = 0.0004) and *CXCL8* (P = 0.0003) mRNA expression compared with vehicle control treatment (Fig. 5D–F). However, addition of nifedipine did not reduce this upregulation of *IL-1B, IL-6,* or *CXCL8* (Fig. 5D–F). Co-treatment with the combination of TNF and LPS significantly increased *IL-1B, IL-6,* and *CXCL8* mRNA expression compared with control (P < 0.0001, Fig. 5G–I). However, consistent with the previous findings with the myometrial cell line, addition of nifedipine did not reduce this upregulation (Fig. 5G–I).

**Figure 6:**
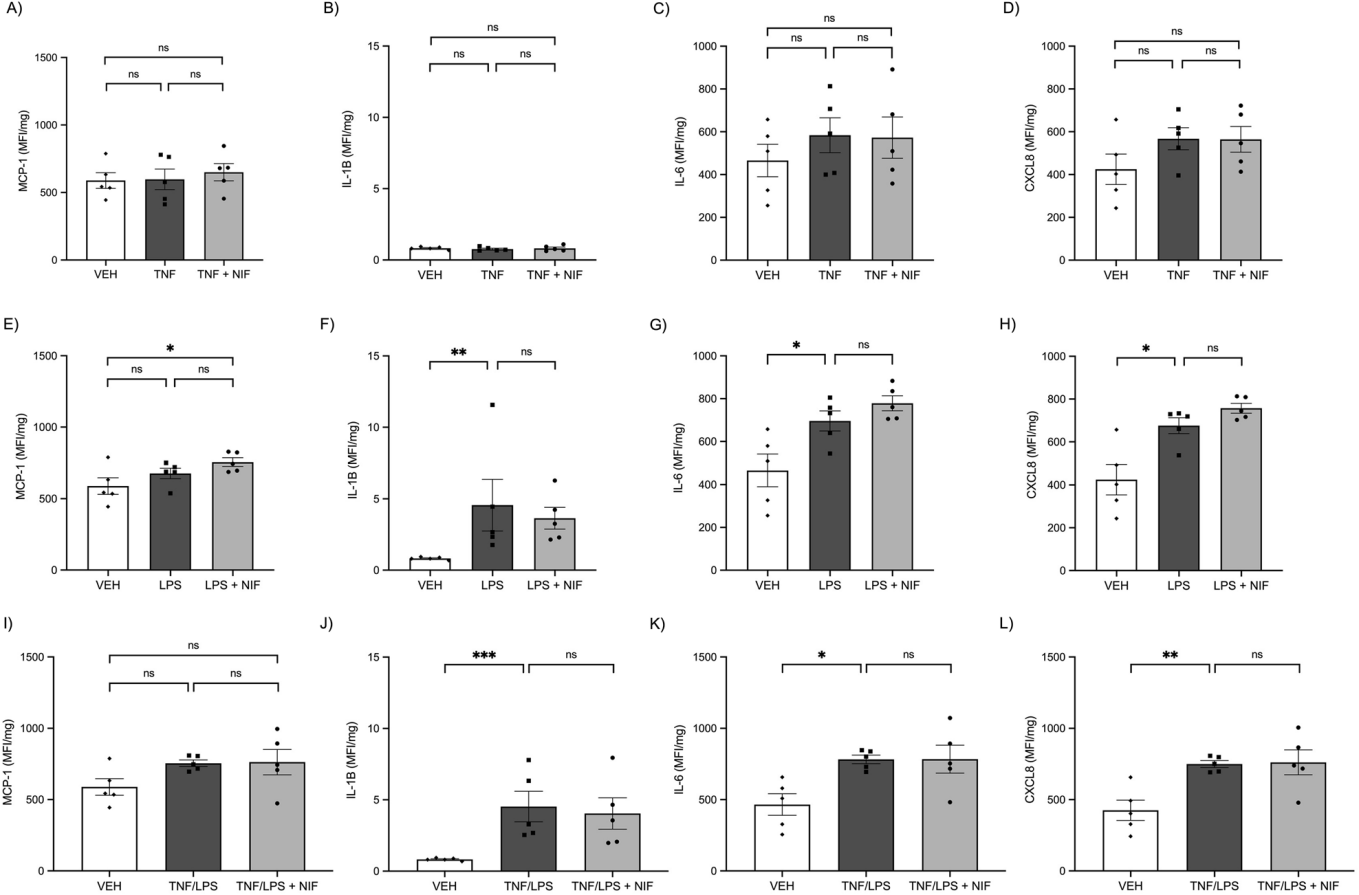
Treatment of human myometrial tissue with (**A–D**) TNF (1 ng/ml), (**E–H**) LPS (5 ng/ml), and (**I–L**) concurrent TNF and LPS, and co-treatment with nifedipine (NIF). TNF does not induce secretion of MCP-1 (**A**), IL-1B (**B**), IL-6 (**C**) or CXCL8 (**D**) compared with vehicle control (VEH). Addition of nifedipine (10 µM) does not have any effect when compared with vehicle. LPS does not increase MCP-1 secretion (**E**), but does induce a significant increase in production and secretion into culture media of IL-1B (**F**), IL-6 (**G**), and CXCL8 (**H**) and co-treatment with nifedipine does not reduce this increased secretion. Concurrent treatment with TNF and LPS does not increase secretion of MCP-1 (**I**) but does increase secretion of IL-1B (**J**), IL-6 (**K**), and CXCL8 (**L**). Data is expressed as the median fluorescence intensity (MFI) of technical replicates as measured by Luminex assay normalised to the mass of tissue (mg). Treatments were performed in duplicate and individual data points represent the mean of those technical replicates (*n* = 5 experiments). Statistical analysis was performed on the log2 transformation of the MFI/mg using an ANOVA followed by Tukey’s multiple comparisons. The error bars represent SEM, * indicates P < 0.05, ** indicates P < 0.01, *** indicates P < 0.001, and ns indicates no statistical difference.

We also investigated if nifedipine could alter the secretion of pro-inflammatory cytokines from myometrial tissue. MCP-3 secretion was below the detectable limit for all experiments. Treatment of myometrial tissue with TNF did not increase secretion of MCP-1, IL-1B, IL-6, or CXCL8 compared with vehicle control treated tissue (Fig. 6A–D). There was also no difference in secretion of these pro-inflammatory cytokines with concomitant nifedipine treatment compared with control (Fig. 6A–D).

Treatment with LPS induced a significant increase in secretion of IL-1B (P = 0.002), IL-6 (P = 0.04), and CXCL8 (P = 0.01) (Fig. 6E–H) compared with control-treated tissue, but LPS treatment did not increase secretion of MCP-1 (Fig. 6). Addition of nifedipine did not alter secretion of MCP-1, IL-1B, IL-6, and CXCL8 induced by LPS treatment (Fig. 6E–H).

Similarly, co-treatment with both TNF and LPS did not induce increased secretion of MCP-1 compared with control-treated tissue, but caused a significant increase in IL-1B (P = 0.0003), IL-6 (P = 0.02), and CXCL8 (P = 0.01) secretion compared with control-treated tissue (Fig. 6I–L). There was no change in the secretion of these cytokines with nifedipine co-treatment (Fig. 6I–L).

### Mouse preterm birth study

**Figure 7:**
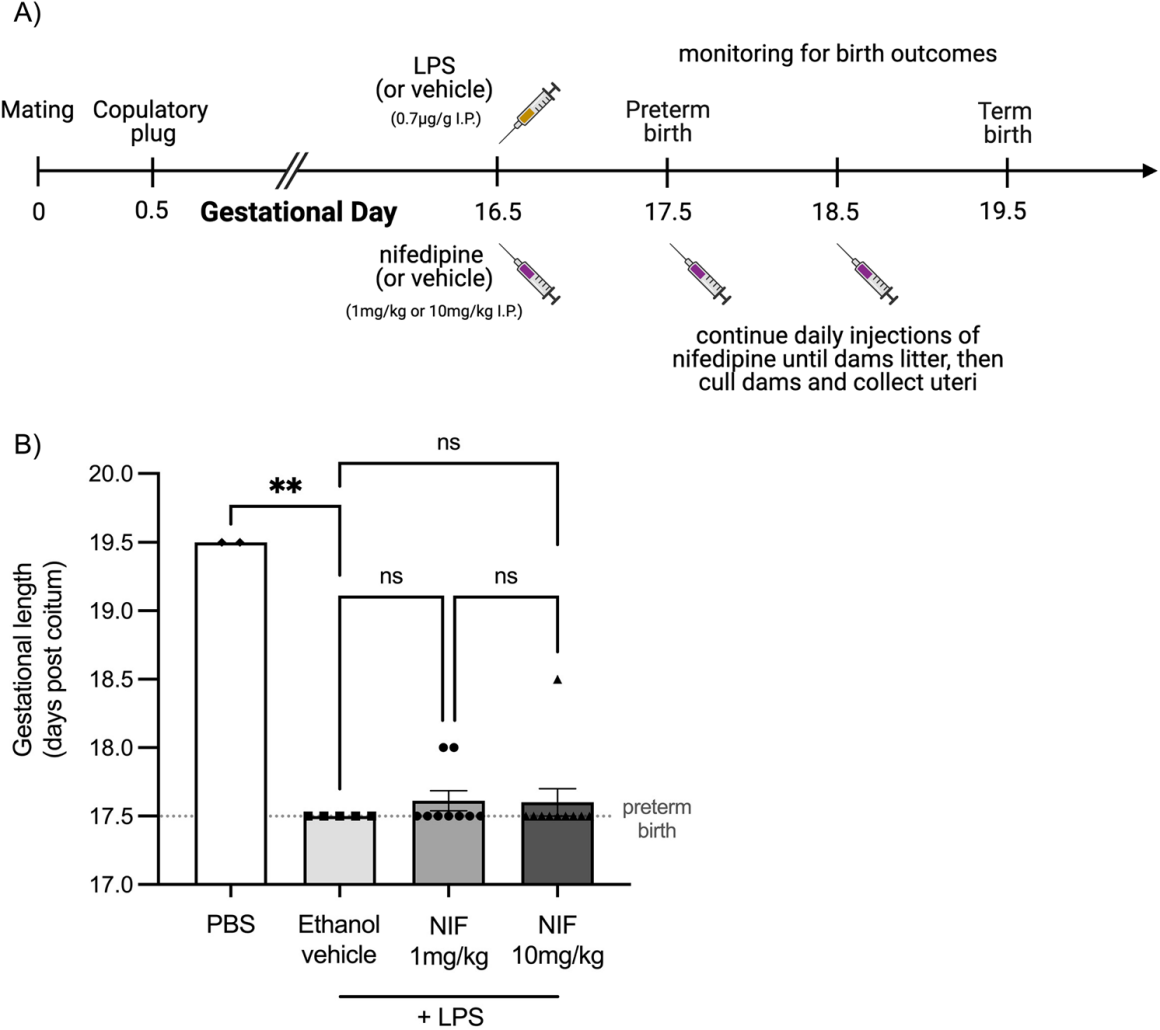
Mice treated with nifedipine (NIF) delayed LPS-induced preterm birth in some mice but was not consistent. (**A**) Timeline indicating the treatment time-points. Pregnant mice were injected intraperitoneal with lipopolysaccharide (LPS; *n* = 24) or vehicle (phosphate-buffered saline; PBS; *n* = 2) on gestational day (D)16.5. Mice were then immediately administered either vehicle (ethanol; *n* = 5), 1 mg/kg nifedipine (n = 9) or 10 mg/kg nifedipine (*n* = 10). Treatments were repeated every 24-h until birth. (**B**) Gestational length was calculated as days post coitum. Each data point represents one dam and error bars represent SEM, ns denotes no significant statistical difference between groups, and ** denotes P < 0.01.

We finally assessed whether nifedipine treatment could prevent preterm birth in mice (Fig. 7A). In pregnant mice, intraperitoneal administration of LPS on D16.5 induced preterm birth (within 24 h) in all mice, compared to the PBS control mice (normal pregnancy) which all delivered at term (D19.5, Fig. 7B). When the mice were treated with nifedipine at either 1 mg/kg or 10 mg/kg, there was no overall significant reduction in preterm birth. However, nifedipine treatment did prolong pregnancy in two mice (treated with 1 mg/kg nifedipine) by 12 h and one mouse (treated with 10 mg/kg nifedipine) by 24 h, but this effect was not statistically significant (Fig. 7B).

We also assessed whether the uteri post-delivery had altered expression of genes associated with inflammation or uterine contraction. There were no differences in expression of *Il-1b, Il-6, Tnf, NLR family pyrin domain containing 3 (Nlrp3), gap junction alpha-1 (Gja1), oxytocin receptor (Oxtr),* and* prostaglandin-endoperoxide synthase 2 (Ptgs2)* between vehicle-treated mice, nifedipine-treated mice with no delay in delivery, and nifedipine-treated mice with delayed delivery (gestation longer than 17.5 days) (Supplementary Fig. S6). However, *C–C motif chemokine ligand 2* (*Ccl2*) expression was lower in nifedipine-treated mice that had a delayed delivery compared with vehicle-control mice and with nifedipine-treated mice that had a preterm delivery (Supplementary Fig. S6F).

## Discussion

This study used a novel pipeline of in vitro*, *ex vivo, and murine in vivo functional models of myometrial contraction and inflammation to assess the actions of nifedipine on myometrium. We have shown that nifedipine at these selected doses reduces inflammation-induced, oxytocin-induced, and spontaneous myometrial contractions in human preclinical models. However, nifedipine does not reduce inflammatory marker expression or secretion of inflammatory cytokines from human myometrial cells and tissues. Additionally, we have shown that nifedipine is unable to consistently delay delivery in a LPS mouse model of preterm birth.

Here we have demonstrated that nifedipine acutely inhibited myometrial contractions and maintains prolonged inhibition in isolated myometrial cells. The effect of nifedipine was potent and near instantaneous. These results support initial preclinical studies showing that nifedipine reduced in vitro myometrial contractility (spontaneous and oxytocin-induced) and in vivo uterine activity in non-pregnant and pregnant patients^4,7,8^. However, those previous preclinical studies assessed the ability of nifedipine to reduce uterine contractions but did not examine any other mechanisms of actions or effects of nifedipine on the myometrium, particularly associated with inflammatory pathways. Here, our study provides a more thorough evaluation of nifedipine, particularly on the key inflammatory response that underpins preterm birth pathophysiology.

In our study, we assessed whether myometrial contraction can be induced in response to pro-inflammatory agents, TNF and LPS. Indeed, we were able to demonstrate that mimicking a pathological inflammatory environment with TNF and LPS induced myometrial cell contraction, which has been shown once previously^15^. However, while these results strengthen the idea that targeting inflammation may be therapeutically efficacious in preventing preterm labour, further work is required.

Myometrial inflammation can be either sterile (in the absence of pathogens) or microbial-associated and tends to vary across gestation^16^. TNF and LPS have been used here to examine the activation of pathways involved in both sterile and microbial inflammation, respectively. LPS consistently and robustly increased myometrial cellular and tissue inflammation, significantly upregulating pro-inflammatory cytokines (mRNA and protein). In response to pathogenic molecular components like LPS, myometrial cells produce cytokines such as IL-1B, IL-6, CXCL8, and TNF which is enhanced by infiltrating leukocytes such as macrophages, thus promoting a regulatory positive feedback loop to maintain myometrial contractility^9^. Nifedipine did not reduce any marker of inflammation we investigated, suggesting that its inhibitory action on smooth muscle contractions is not via regulation of pro-inflammatory cytokines. Conversely, TNF exposure for six hours did not induce expression or production of key pro-inflammatory cytokines. This may be a timing effect, but of note, LPS induced acute pro-inflammatory upregulation in the same time-frame, and as such may be due to inherent differences in mechanisms of action.

There is evidence in other tissues that nifedipine may have anti-inflammatory properties beyond calcium channel inhibition. For example, in human osteoarthritic chondrocytes, nifedipine at a concentration similar to the one used here inhibited expression of IL-1B, IL-6, TNF, and cyclooxygenase-2, as well as inhibiting oxidative stress^13^. Additionally, nifedipine is believed to have anti-inflammatory and anti-oxidative stress effects on endothelial cells^14^. Therefore, while it was possible that nifedipine could have an anti-inflammatory effect on myometrial smooth muscle cells and tissue, results from this study do not support this hypothesis.

To recapitulate inflammation associated with systemic infection, a known trigger of premature activation of the myometrium, preterm labour was induced in an in vivo mouse model using LPS. In our model, all mice gave birth preterm within 24 h of receiving LPS. This consistent timing of preterm birth ensured that any observed delay in pregnancy was due to nifedipine and not to a variable effect of LPS. Nifedipine was unable to consistently prolong gestation in this model, which contrasts to what is observed clinically, where a recent randomised control trial showed that nifedipine treatment delayed preterm birth by 48 h in almost 80% of cases^17^. However, while it is difficult to determine the exact equivalent timeframe, the 12 to 24 h delay in the mouse gestation observed in this study can be approximated to be a few days to a week in human pregnancy. Therefore, this mouse model can recapitulate preterm birth, but does not completely mirror the effects of nifedipine seen in humans in the clinic. This may be due to the differences between human and mouse parturition which is a limitation of this study. This highlights the benefits of developing and employing a multi-faceted experimental approach as used in our study.

The mRNA expression of pro-inflammatory or contraction-associated genes in the mouse uteri post-birth did not differ between control mice that delivered preterm or mice that received nifedipine. However, *Ccl2* expression was downregulated in uteri from the dams in which nifedipine delayed the deliveries. Strong conclusions cannot be drawn, however it is known in humans that CCL2 (also known as MCP-1) is upregulated by the myometrium during labour^18^ so this raises interest in whether CCL2 could stand as an attractive candidate to target for a therapy. To note, a limitation of the current study is that as the time between delivery of the pups and collection of the uteri was not controlled for within the mouse cohort. To gain a better understanding of how these gene profiles may differ with nifedipine treatment, the precise time of birth would be informative to ensure similar time between mouse uteri directly following labour.

Our study provides evidence that nifedipine does not reduce inflammation that likely drives uterine contractions via upstream pathways. This is the first study to investigate the potential effect of nifedipine on pro-inflammatory pathways in human myometrium, an important consideration for any potential tocolytic. Without anti-inflammatory effects, nifedipine may not provide protection to the fetus against the detrimental effects of preterm labour-associated inflammation, but could be combined with an anti-inflammatory agent for dual therapy. Therefore, in cases where nifedipine is used to delay delivery, this must be considered and highlights the urgent need for effective therapeutics that can safely prolong gestation and quench inflammation.

In our preterm birth mouse model, whereby preterm mouse fetuses are not viable and difficult to collect, we were unable to assess if nifedipine had any effects in improving inflammation in utero. Future studies could investigate earlier timepoints of gestation post-nifedipine treatment to determine if there were still detrimental inflammatory effects on the fetuses, particularly on the fetal brain. A dual therapy of nifedipine and a known anti-inflammatory agent could also be investigated to determine if this counters the inflammation associated with preterm labour. To complement our work in the human myometrium, investigation of the effect of nifedipine on human fetal membranes, the decidua and the placenta should be investigated to gain a more holistic understanding of nifedipine’s action on all the gestational tissues that work in unison to maintain uterine quiescence and maternal immune tolerance during pregnancy.

The major strength of this study is the inclusion of multiple in vitro, ex vivo, and in vivo models, which in unison form a robust, multi-faceted research approach. The studies used in here build upon and enhance already established experimental protocols giving the methodology of this study credibility. However, our adaptation of the Danish Myo Technology (DMT) myograph to measure human myometrial tissue in this study is innovative, highly sensitive and a superior method to traditional tissue bath set-ups. Previous studies in the preterm birth research space often rely on a single model to assess the effects of innovative therapies. Our pipeline allowed the study of the effects of nifedipine at the cellular, whole tissue, and whole systems levels.

One consideration to note is the use of myometrial samples obtained from term pre-labour pregnancies and not from preterm labouring pregnancies. Therefore, the findings from this study may not translate directly to clinical settings and may not reflect the same effect in labouring myometrium. However, by limiting our sample collection to term pregnancies we were able to study the in vitro effects of nifedipine in a homogenous and physiologically normal study population. Additionally, in using oxytocin within the tissue baths, we could simulate some conditions of labour.

When utilising whole myometrial tissue collected from patients, we cannot conclude that the effects are caused directly by the main functional cells of the myometrium—the smooth muscle cells—but could be due to multiple cell types working in concert. Additionally, the myometrial samples collected from the lower segment of the uterus during caesarean section does not uniformly represent all the cellular and tissue structures that the entire uterus is composed of^19^. Further work could elucidate the contributions of the other cell types in regulating the inflammatory response.

We have demonstrated nifedipine blocks both spontaneous and, for the first time, inflammation-induced uterine contraction in vitro and ex vivo. However, as preterm labour is a complex process, inhibiting muscular contraction of the uterine myometrium may not be enough to prevent preterm birth. We have shown that nifedipine does not reduce markers of inflammation and may explain why nifedipine is unable to consistently perform as a tocolytic clinically. Therefore, nifedipine appears to treat the symptoms of preterm labour rather than the cause, thus is limited as a preterm birth therapy. Our study highlights the crucial need for new therapeutics for preterm birth that target the inflammatory pathways upstream of myometrial contractions.

## Methods

### Drugs and chemicals

Nifedipine (N7634, Sigma-Aldrich, Missouri, USA) was reconstituted in sterile ethanol with vortexing at 50 mM and was stored in a parafilm-sealed tube (to prevent evaporation) in the dark at 4 °C. *E. Coli* lipopolysaccharide (LPS; L2630, Sigma) was reconstituted in Dulbecco’s phosphate-buffered saline (dPBS; Gibco, ThermoFisher Scientific, Victoria, Australia), aliquoted and stored at − 20 °C. Recombinant human tumour necrosis factor alpha (TNF; Gibco; ThermoFisher Scientific) was reconstituted in sterile water, aliquoted and stored at − 80 °C. L-glutamine was prepared by dissolving 73 mg L-glutamine (Sigma-Aldrich) in sterile water then filter sterilising to make a 100 × stock solution for 1:100 dilution into media. TSL was prepared by dissolving 10 ug/ml transferrin, 25 ng/ml sodium selenite, 10 nmol/l linoleic acid in sterile water.

### Human myometrium tissue collection

Ethical approval for this study was obtained from the Mercy Health Human Research Ethics Committee (2020-051). All methods were performed in accordance with the National Health and Medical Research Council ethical guidelines. Pregnant individuals presenting to the Mercy Hospital for Women, Heidelberg, Australia, gave informed written consent for myometrial tissue collection. A two-to-three-centimetre diameter myometrial sample was excised from the lower portion of the non-labouring (and non-induced) uterus at term caesarean sections, from *n* = 11 singleton pregnancies with no known complications, no prior history of preterm birth, and no medication use during pregnancy. Patient characteristics and demographic data are shown in Table 1. Samples were collected into cold phosphate buffer saline (PBS; 137 mM NaCl, 10 mM Na_2_HPO_4_, 1.8 mM KH_2_PO_4_, 2.7 mM KCl, pH 7.4) and processed within 30 min of collection.

**Table 1:** Demographic characteristics of the pregnant patients.

Characteristics	Value
Maternal age (years)	32.8 ± 2.9
Maternal body mass index pre-pregnancy (kg/m^2^)	21.7 ± 7.8
Maternal body mass index at delivery (kg/m^2^)	28.5 ± 4.2
Gestational age (weeks)	38.6 ± 0.4
Fetal body weight at birth (g)	3565 ± 378
Ethnicity	
Caucasian (Europe, Middle East, North Africa, Americas, Australia)	8 (72.7)
East Asian (China, Korea, Japan, South East Asia)	1 (9.1)
Central Asian (India, Pakistan, Nepal, Sri Lanka)	2 (18.2)
Gravidity n	
1	0 (0)
2	3 (27.3)
3	3 (27.3)
4	2 (18.2)
5	2 (18.2)
6	1 (9.1)
Parity n	
1	2 (18.2)
2	3 (27.3)
3	3 (27.3)
4	2 (18.2)
5	1 (9.1)

Continuous variables are shown as the mean ± standard deviation; categorical variables are shown as the number of cases (%).

### Human myometrial strip contractility myography

Myometrial tissue samples were transferred to cold Krebs buffer (120 mM NaCl, 5 mM KCl, 1.2 mM MgSO_4_, 1 mM KH_2_PO_4_, 25 mM NaHCO_3_, 11.1 mM D-Glucose, 2.5 mM CaCl_2_) and dissected with a scalpel into strips 8 mm long x 2 mm wide x 1 mm thick along the longitudinal axis aligned with the direction of the muscle fibres. Strips were mounted to individual organ baths (820MS system, Danish Myo Technology, Hinnerup, Denmark) filled with 7 mL Krebs buffer. Within each organ bath, one end of the muscle strip was clamped to a calibrated force transducer and the other end to a micromanipulator, so that the tissue between the clamps was ~ 5 mm long. Once myometrial strips were clamped into the bath, a passive tension of 2mN was applied^20^. Each organ bath was continuously aerated with carbogen (95% O_2_, 5% CO_2_) and maintained at a temperature of 37 °C. Data were collected and analysed using LabChart Pro Version v8 1.21.

Spontaneous rhythmic contractions typically initiated within two hours of mounting the tissue strips. Tissue strips that failed to develop spontaneous contractions within two hours were challenged with a high potassium salt solution (40 mM KPSS; 85 mM NaCl, 40 mM KCl, 1.2 mM MgSO_4_, 1 mM KH_2_PO_4_, 25 mM NaHCO_3_, 11.1 mM D-Glucose, 2.5 mM CaCl_2_) for two minutes. If the tissue was non-responsive, it was removed from the bath and replaced with a new strip in fresh Krebs buffer and then left to equilibrate and develop spontaneous contractions.

In an additional set of experiments, spontaneously contracting myometrium was further stimulated with 1 nM oxytocin in the bath to amplify contractility, based on the method of Arrowsmith and colleagues (2018) and optimised for this assay^20^.

For each myometrial strip, a pre-treatment contraction baseline (one hour of stable basal contractions) was established to serve as reference. Nifedipine stock solution (50 mM in ethanol) or vehicle (ethanol) was diluted 1:500 in Krebs buffer to an intermediate concentration of 100 µM. Then, 7 µL of this intermediate solution was added to 7 mL buffer in the baths, to perform a 1:1000 dilution producing a final concentration of nifedipine at 0.1 µM and a final concentration of 0.0002% v/v ethanol. Dose response experiments were performed to determine the optimal concentration that elicits an inhibitory effect and prevents any effect of the ethanol as vehicle. Myometrial strips were incubated with nifedipine or vehicle for 1 h. Myometrial strips were then washed thrice with fresh 37 °C Krebs buffer to remove all treatment (washout) and then allowed to re-equilibrate for one hour before being challenged with 40 mM KPSS to check responsiveness at completion of experiment.

To determine changes in myometrial contractility, the frequency (contractions per hour), amplitude (height of peak), time to peak (time from initiation of contraction to max amplitude), and duration (width of single contractions) of contractions were measured using LabChart (v8, ADInstruments, Bella Vista, NSW, 2153, Australia). Changes in these outputs were normalised to the basal (pre-treatment) spontaneous contractions of each strip: the frequency, amplitude, time to peak and duration in the pre-treatment period were assigned 100% and these outputs in the post-treatment period were calculated as a percentage of these baseline contractions. This relative difference was then calculated relative to the baseline contractions of the pre-treatment vehicle-treated strips.

### Human myometrial smooth muscle cell line culture and expansion

An immortalised myometrial smooth muscle cell line, Pregnant Human Myometrial 1–41 (PHM1-41, ATCC, Virginia, USA), was cultured in T75 flasks containing Dulbecco’s modified eagle medium (DMEM; High glucose + Glutamax, Gibco; ThermoFisher Scientific) supplemented with 10% fetal calf serum (FCS; Gibco; ThermoFisher Scientific), 2 mM L-glutamine, and 0.1 mg/ml Geneticin (G-418; Roche Diagnostics, Victoria, Australia). Cells were expanded and used no higher than passage 30 for in vitro experiments.

### Human myometrial smooth muscle cell contraction assay

Collagen gel contraction assays were performed to assess the effect of nifedipine on the contractility of myometrial smooth muscle cells. Confluent PHM1-41 cells were harvested with TryplE Express Enzyme (Gibco; ThermoFisher Scientific), centrifuged at 150xg for 5–10 min and resuspended in serum-free DMEM containing 1% TSL and 2 mM L-glutamine.

Cultrex rat collagen I (R&D Systems, Minnesota, USA) was combined with sterile 10X dPBS, water, and 1N sodium hydroxide on ice as described in the manufacturer’s manual to make a 3 mg/ml collagen solution. Stock TNF, LPS and nifedipine were diluted in their vehicles (water, PBS, and ethanol, respectively). Vehicles of each agonist were accounted for in each treatment.

Cells at a density of 150,000 cells/ml were added to the collagen solution in a 2:1 ratio. To stimulate contractions, 1 ng/ml TNF was added into this cell/collagen solution. Nifedipine (10 µM) or vehicle control (ethanol) was also added into the solution. Dose response experiments were used to determine the optimal concentration of nifedipine to elicit an inhibitory response but was not cytotoxic. 500 μL of this cell/collagen mixture was pipetted into wells of 24-well plates. The cells were embedded in the collagen gel by incubating the plate at 37 °C for one hour until the gels had solidified into discs. Then, 500 μL of serum-free media containing 100 ng/ml LPS and 10 µM nifedipine (or vehicle control) treatments were added to the wells. The gel discs were detached from the well walls by gently running a pipette tip along the gel edges. The plates were incubated for 48 h. A cell-free collagen-gel only experiment was also performed to determine if there was any contraction inherent to the collagen gel itself.

Images of the floating gel discs were taken at 0 h and 48 h using a ChemiDoc Imaging System (BioRad, California, USA). The size of the gels was determined by the area as measured using ImageJ software (v1.53a, National Institute of Health, Maryland USA). The gel area measurements at 48 h were calculated as a percentage original area of the gels at the initial timepoint (0 h) for each independent experiment. Treatments were performed in quadruplicate and the experiment was repeated thrice.

### Myometrial cell inflammatory response studies

PHM1-41 cells were seeded at a density of 40,000 cells/well into 24-well plates in DMEM supplemented with 10% FCS and 2 mM L-glutamine (G-418 omitted as per ATCC recommendation). Cells were incubated overnight at 37 °C (5% CO_2_, 20% O_2_) to adhere to the plate. Cells were then serum-starved by replacement of media with DMEM supplemented with 1% TSL and 2 mM L-glutamine and incubated for a further 16 h. Following serum-starving, cells were pre-treated with either TNF (0.1 ng/ml), LPS (100 ng/ml) or vehicle control in a low-serum media (DMEM supplemented with 2% FCS and 2 mM L-glutamine) for two hours. Subsequently, this pre-treatment was removed and replaced with treatments of TNF or LPS, with or without nifedipine (10 µM) and incubated for another 24 h. At completion, cells were washed with PBS and frozen at − 80 °C with lysis solution (Sigma-Aldrich) in preparation for RNA extraction.

### Cell viability assay

Cell viability was assessed for each dose of TNF, LPS, and nifedipine used. The MTS assay (Promega, Madison WI, USA) was carried out in a 96-well plate as per manufacturer’s instructions. Optical density at 490 nm absorbance was measured using a BioRad X-Mark Microplate Spectrophotometer and Bio-Rad Microplate Manager 6 software.

### Ex vivo myometrial tissue inflammatory response

Myometrial tissue samples were dissected into fragments of approximately 1–2 mm size with any vasculature, decidua or scar tissue excised and excluded. Three pieces of dissected tissue (combined weight 20–30 mg) were placed into each well of a 24-well plate containing treatments of combinations of TNF (1 ng/ml), LPS (5 ng/ml), and nifedipine (10 µM) or vehicle in DMEM (supplemented with 1% antibiotic–antimycotic (Gibco; ThermoFisher Scientific), 10% FCS, and 2 mM L-glutamine). Myometrial tissue was incubated at 37 °C under 8% O_2_ and 5% CO_2_ for 6 h. The tissue pieces were collected, blotted, weighed, and stored in RNAlater at 4 °C for 48 h before snap-freezing in liquid nitrogen and storage at − 80 °C.

### Cytokine secretion assay

A panel of human pro-inflammatory cytokines and chemokines (IL-1β, IL-6, IL-8 (CXCL8), monocyte chemoattractant protein (MCP)-1 and MCP-3) were simultaneously measured (multiplex) in the collected cell and tissue media supernatant using Milliplex MAP Luminex microbead assays (Merck Millipore, Massachusetts, USA) as per manufacturer’s instructions. Technical duplicates from each cell culture experiment were pooled prior to multiplexing. Samples were multiplexed in technical duplicate without dilution and data analysed on a Luminex (Bioplex-200, BioRad) instrument. Minimum detectable thresholds were 0.8 pg/ml (IL-1β), 0.9 pg/ml (IL-6), 0.4 pg/ml (IL-8), 1.9 pg/ml (MCP-1), and 3.8 pg/ml (MCP-3). For evaluation of protein expression by myometrial smooth muscle cells in vitro results are expressed as mean fluorescent intensity. For protein expression of myometrial tissue cultured and treated ex vivo, the mean fluorescence intensity was calculated per milligram of tissue cultured to control for differences in tissue weight.

### Mouse PTB model

Animal experiments were approved by the Austin Health Animal Ethics Committee (A2020/05672) and followed the National Health and Medical Research Council ethical guidelines for the care and use of animals for scientific purposes. All methods have been reported in accordance with the ARRIVE guidelines (https://arriveguidelines.org). Five-week-old CBA x C57BL/6 (F1) female mice (*n* = 26) were sourced from Animal Resources Centre (Western Australia, Australia). Mice were group-housed in conventional open-top cages (18–22 °C; 50% relative humidity), with a 12-h light/dark cycle, and food and water available ad libitum. Female mice were acclimated to the new facility for 1 week before being mated overnight with stud F1 male mice. Pregnancy was confirmed by the presence of a copulatory plug the following morning, designated as gestational day (D)0.5. On the morning of D16.5, pregnant mice (*n* = 24) received a 100 µl intraperitoneal injection of 0.7 µg/g LPS in PBS to induce preterm delivery. Mice were randomised and immediately following LPS injection, mice received either a 20 µl intraperitoneal injection of 1 mg/kg (*n* = 9) or 10 mg/kg (*n* = 10) nifedipine in ethanol or neat ethanol as vehicle control (*n* = 5) (See Fig. 7A). The gestational length (from mating to birth) was recorded for each dam. Mice that had littered by the morning of D17.5, within 24 h of LPS administration, were considered to have delivered preterm. A subset of mice (*n* = 2) received only a 100 µl intraperitoneal injection of PBS on D16.5 as control for term gestation length in these mice.

Dams were humanely killed via cervical dislocation the morning after they littered. Both uterine horns were dissected and a portion of each horn was collected into RNAlater. Uterine horns were stored at 4 °C in RNAlater for a minimum of 48 h before snap freezing in liquid nitrogen and storage at − 20 °C. Investigators were not blinded when administering I.P. injections, but were blinded when analysing gestational length of each mouse.

### Reverse transcription and qRT-PCR

Total RNA was extracted from cells, tissue, and mouse uterine horns using the GenElute Mammalian Total RNA Miniprep Kit (Sigma-Aldrich) according to the manufacturer’s instructions. Myometrial tissue and mouse uterine horns (maximum 40 mg) were homogenised using a tissue homogeniser (Omni International, Georgia, USA) prior to RNA extraction and a proteinase K digestion (P4850, Sigma-Aldrich) was included. RNA was quantified using the Nanodrop ND 1000 spectrophotometer (Nanodrop Technologies Inc, Delaware, USA) and then converted to cDNA using the High Capacity cDNA Reverse Transcription Kit (Applied Biosystems, Massachusetts, USA) as per manufacturer’s guidelines.

Quantitative real-time polymerase chain reaction (qPCR) was performed to evaluate the effect of TNF, LPS, and nifedipine treatment on expression of pro-inflammatory and myometrial contraction-associated genes. Predesigned TaqMan gene expression assays were used to quantify mRNA expression of these genes of interest (listed in Table 2). Gene expression was quantified by real time qPCR on the CFX384 (BioRad) using FAM-labelled Taqman universal PCR mastermix (Applied Biosystems) with the following thermocycling conditions: 50 °C for 2 min; 95 °C for 10 min, 95 °C for 15 s, 60 °C for 1 min (40 cycles).

**Table 2:** TaqMan gene expression assay IDs.

Gene	Full name	Species	TaqMan ID
*YHWAZ*	Tyrosine 3-monooxygenase/Tryptophan 5-monooxygenase activation protein zeta	Human	Hs01122454_m1
*TOP1*	DNA topoisomerase I	Human	Hs00243257_m1
*IL-1β*	Interleukin-1 beta	Human	Hs01555410_m1
*IL-6*	Interleukin-6	Human	Hs00174131_m1
*CXCL8*	CXC motif chemokine ligand-8	Human	Hs00174103_m1
*Polr2a*	RNA polymerase II subunit A	Mouse	Mm00839502_m1
*Il-1b*	Interleukin-1 beta	Mouse	Mm00434228_m1
*Il-6*	Interleukin-6	Mouse	Mm00446190_m1
*Tnf*	Tumor necrosis factor	Mouse	Mm00443258_m1
*Nlrp3*	NLR family pyrin domain containing 3	Mouse	Mm00840904_m1
*Gja1*	Gap junction alpha-1	Mouse	Mm00439105_m1
*Ccl2*	C–C Motif chemokine ligand 2	Mouse	Mm00441242_m1
*Oxtr*	Oxytocin receptor	Mouse	Mm01182684_m1
*Ptgs2*	Prostaglandin-endoperoxide synthase 2	Mouse	Mm00478374_m1

All cDNA samples were run in technical duplicates in the PCRs. Data were normalised to house-keeping genes (*YHWAZ* for myometrial cells, *TOP1* for primary myometrial tissue, and *Polr2a* for mouse uteri) as internal controls. The stability of these reference genes was confirmed for each different tissue type and has been previously examined^21^. Ct data were analysed using the ΔΔCt method of analysis. Statistical analysis was performed on the ΔCt values and data were then calculated and graphed as fold-change relative to the agonist treatment (i.e. TNF or LPS) using the 2^−ΔΔCt^ method.

## Supplementary Information

Supplementary Figures.

## Data Availability

The datasets generated and analysed in the current study are available from the corresponding author on reasonable request.
