# Supplementary material for: Assessment of the tocolytic nifedipine in preclinical primary models of preterm birth
Source: Sci Rep. 2023 Apr 6;13:5646. doi: 10.1038/s41598-023-31077-x (PMC10079980; doi:10.1038/s41598-023-31077-x)
Supplement: Supplementary file 1 — Supplementary Figures. [file 41598_2023_31077_MOESM1_ESM.docx]

**Supplementary figures**

**
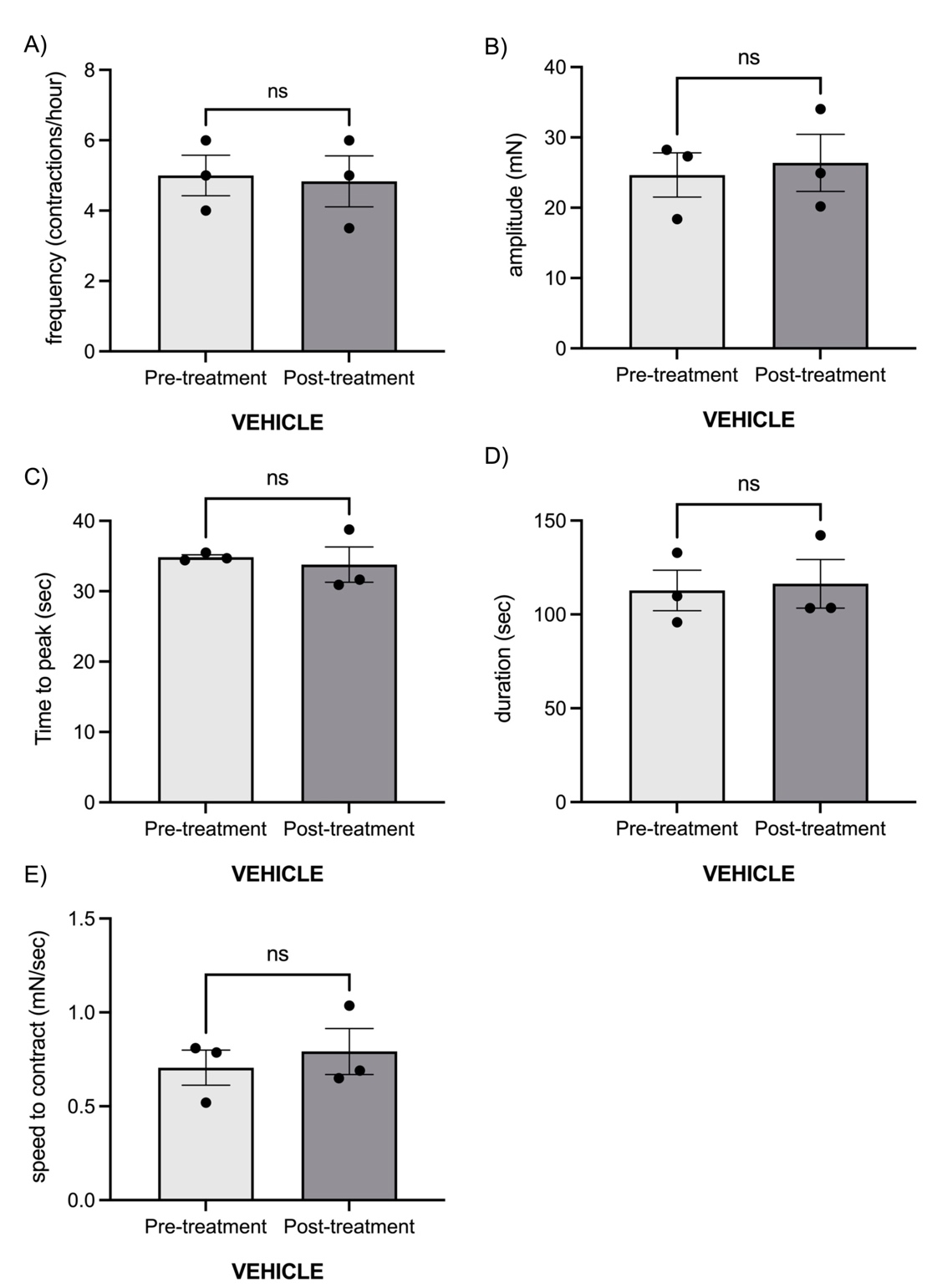
**

**Figure S1:** Vehicle (ethanol) has no effect on established spontaneous contractions of human non-labouring myometrial strips on contraction A) frequency, B) amplitude, C) time to peak, D) duration, or E) speed. Each point represents the mean of duplicates (*n*=3 patients). Differences were analysed with paired t tests. The error bars represent SEM and ns denotes no statistical difference.  ​

**
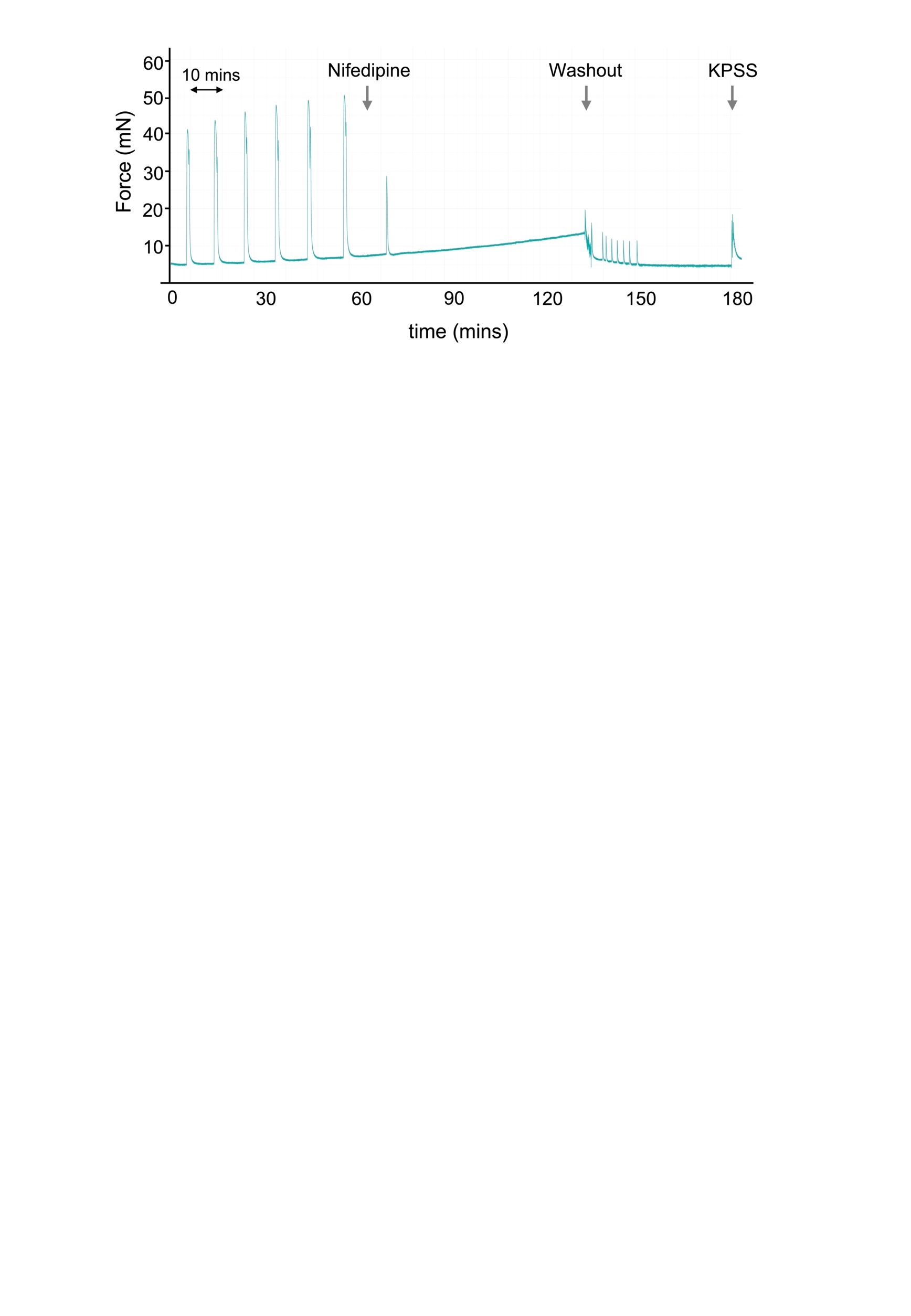
**

**Figure S2:** Representative trace showing contractile activity of nifedipine-treated myometrium after treatment washout and response to final challenge with a high potassium salt solution (KPSS). The y-axis presents the measured force (millinewtons; mN). The entire initial 120 minute equilibration period is not shown in this representative trace.

**
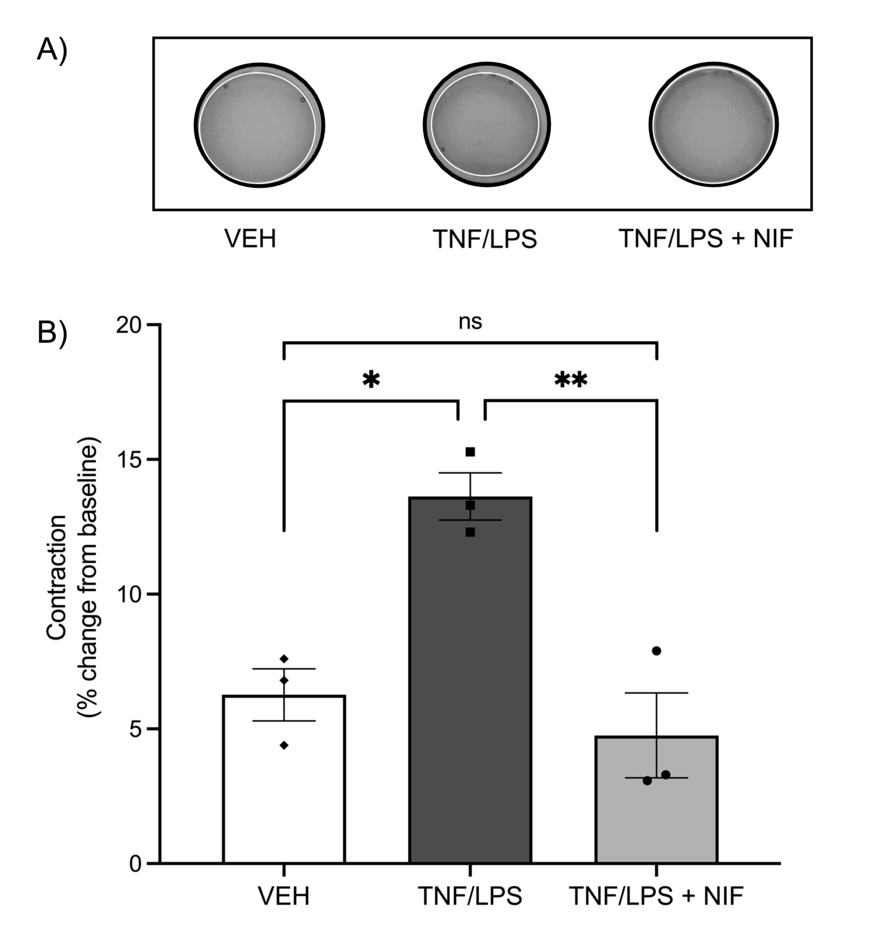
**

**Figure S3:** Treatment with TNF (1ng/ml) and LPS (100ng/ml) in combination induced contraction of myometrial smooth muscle cells embedded in collagen gel compared with vehicle control (VEH) treatment, and nifedipine (NIF) treatment (10µM) inhibits these induced contractions. A) Representative photographs of collagen gel discs containing myometrial cells 48 hours post-treatment, with perimeters of gel discs outlined in white. B) Contraction of cells 48 hours post-treatment relative to baseline at 0 hours. The areas of the gel discs were measured at 0 hours and 48 hours and the percent decrease in gel area was calculated by subtracting the final size from the original size. A decrease in the size of the gel discs indicates contraction of cells. Treatments were performed in quadruplicate and individual data points in this figure represent the mean of those technical replicates (*n*=3 experimental replicates). Data were assessed for statistical differences using ANOVA followed by Dunnett’s multiple comparisons. The error bars represent SEM,  * indicates P<0.05, ** indicates P<0.01, and ns indicates no statistical difference.

**
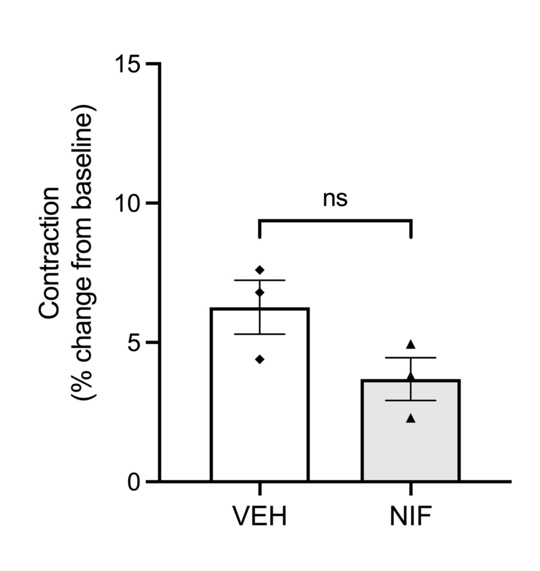
**

**Figure S4:** Nifedipine-only treatment of myometrial cells (PHM1-41 cell line) embedded in collagen gel did not induce contractions. Cells were treated with either vehicle control (ethanol) or nifedipine (10µM) to determine if nifedipine itself has an effect on myometrial contraction. The sizes of the collagen gels were measured at 0h and 48h and the percent decrease in gel area was calculated by subtracting the final size from the original size. A decrease in the size of the collagen gel discs would indicate contraction of cells. Treatments were performed in quadruplicate and individual data points in this figure represent the mean of those technical replicates (*n*=3 experimental replicates). The error bars represent SEM and ns indicates not-significantly different.

**
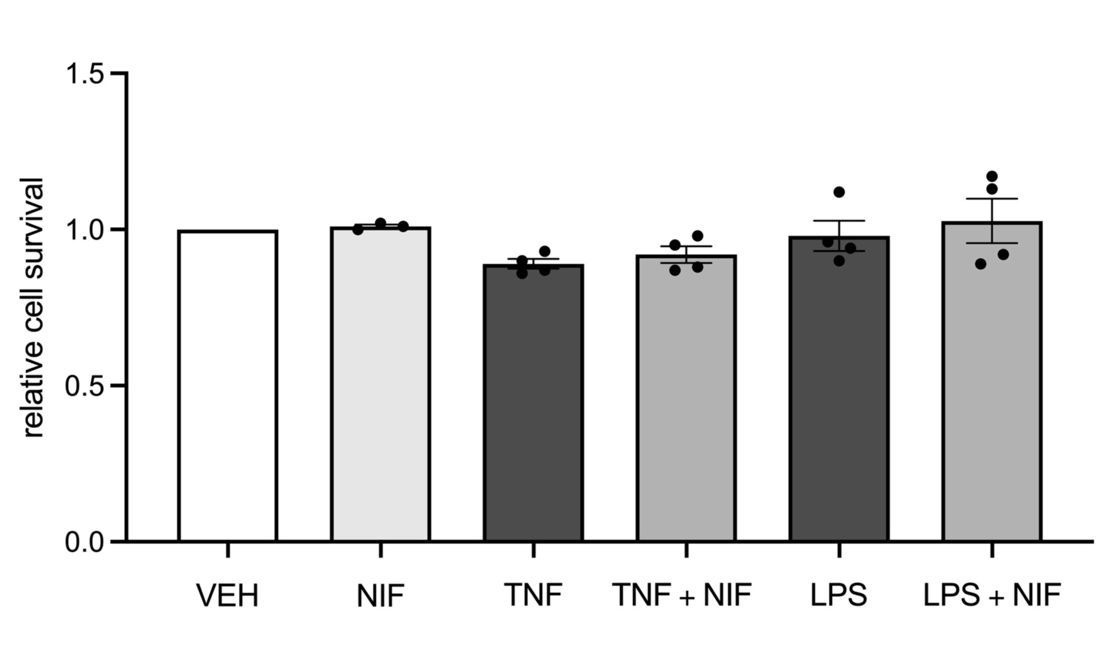
**

**Figure S5:** Treatment with nifedipine (NIF; 10µM), TNF (0.1ng/ml), LPS (100ng/ml), or a combination of these treatments did not affect cell viability as assessed by MTS assay (*n*=4 experiments). Absorbance was read at a wavelength of 490nm and calculated relative to the absorbance reading of the vehicle-treated (VEH) cells. Differences were analysed using ANOVA followed by Dunnett's multiple comparisons. Cell survival was not statistically different between treatments (P>0.05). The error bars represent SEM.

**
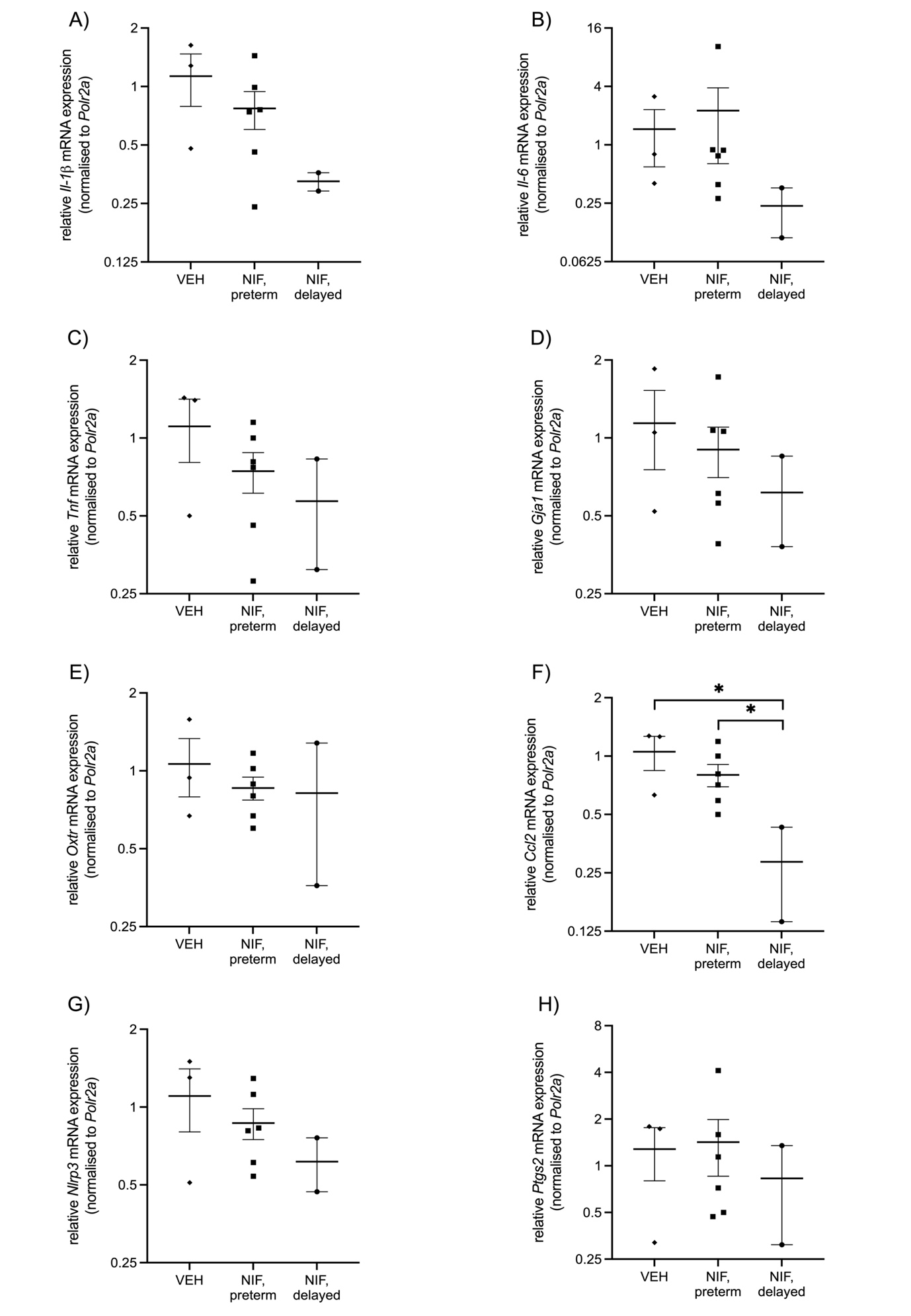
**

**Figure S6:** Gene expression of pro-inflammatory cytokines (A-C, F-H) and contraction-associated proteins (D-E) of mouse uteri collected after delivery. Mice were treated with LPS and then either vehicle (VEH; ethanol) or 1mg/kg nifedipine (NIF). In these graphs, the mice that received nifedipine were stratified depending on whether the mice delivered preterm (gestational length 17.5 days) or if the delivery was delayed (gestational length >17.5 days). Data are presented as fold change calculated relative to that of the vehicle-treated mice. Individual data points represent the mean of technical replicates of uteri from each mouse and the error bars represent SEM. One-way ANOVA followed by Tukey’s multiple comparisons was performed on the deltaCt (Ct of the gene of interest subtracted from the Ct of the *Polr2a* reference gene). No statistical differences were detected between groups (P>0.05) except where indicated with *, representing P<0.05.
